# Identification of a Stable Hydrogen-Driven Microbiome in a Highly Radioactive Storage Facility on the Sellafield Site

**DOI:** 10.3389/fmicb.2020.587556

**Published:** 2020-11-24

**Authors:** Sharon Ruiz-Lopez, Lynn Foster, Chris Boothman, Nick Cole, Katherine Morris, Jonathan R. Lloyd

**Affiliations:** ^1^Department of Earth and Environmental Sciences, University of Manchester (UoM), Manchester, United Kingdom; ^2^Sellafield Ltd., Warrington, United Kingdom

**Keywords:** spent nuclear fuel, microbial ecology, Sellafield, *Hydrogenophaga*, spent nuclear fuel ponds, hydrogen metabolism

## Abstract

The use of nuclear power has been a significant part of the United Kingdom’s energy portfolio with the Sellafield site being used for power production and more recently reprocessing and decommissioning of spent nuclear fuel activities. Before being reprocessed, spent nuclear fuel is stored in water ponds with significant levels of background radioactivity and in high alkalinity (to minimize fuel corrosion). Despite these challenging conditions, the presence of microbial communities has been detected. To gain further insight into the microbial communities present in extreme environments, an indoor, hyper-alkaline, oligotrophic, and radioactive spent fuel storage pond (INP) located on the Sellafield site was analyzed. Water samples were collected from sample points within the INP complex, and also the purge water feeding tank (FT) that supplies water to the pond, and were screened for the presence of the 16S and 18S rRNA genes to inform sequencing requirements over a period of 30 months. Only 16S rRNA genes were successfully amplified for sequencing, suggesting that the microbial communities in the INP were dominated by prokaryotes. Quantitative Polymerase Chain Reaction (qPCR) analysis targeting 16S rRNA genes suggested that bacterial cells in the order of 10^4^–10^6^ mL^–1^ were present in the samples, with loadings rising with time. Next generation Illumina MiSeq sequencing was performed to identify the dominant microorganisms at eight sampling times. The 16S rRNA gene sequence analysis suggested that 70% and 91% from of the OTUs samples, from the FT and INP respectively, belonged to the phylum Proteobacteria, mainly from the alpha and beta subclasses. The remaining OTUs were assigned primarily to the phyla Acidobacteria, Bacteroidetes, and, Cyanobacteria. Overall the most abundant genera identified were *Hydrogenophaga*, *Curvibacter*, *Porphyrobacter*, *Rhodoferax*, *Polaromonas*, *Sediminibacterium*, *Roseococcus*, and *Sphingomonas.* The presence of organisms most closely related to *Hydrogenophaga* species in the INP areas, suggests the metabolism of hydrogen as an energy source, most likely linked to hydrolysis of water caused by the stored fuel. Isolation of axenic cultures using a range of minimal and rich media was also attempted, but only relatively minor components (from the phylum Bacteroidetes) of the pond water communities were obtained, emphasizing the importance of DNA-based, not culture-dependent techniques, for assessing the microbiome of nuclear facilities.

## Introduction

Nuclear power supplies about 11% of the world’s electricity (WNA, 2016), and with increasing global energy demands this seems unlikely to decline. Although considered a “low carbon” generating energy source, radioactive waste is produced, including spent fuels that need storage prior to reprocessing and final disposal (Deutch et al., 2009). In the United Kingdom, this task is performed at Sellafield, one of the largest and most complex nuclear sites in Europe. With over 1,400 discrete operations, handling 240 nuclear materials, it is located in Cumbria on the North West coast of England and has been operated by the Nuclear Decommissioning Authority (NDA) since 2005 (Baldwin, 2003; WNA, 2018a). Calder Hall, located on the site, was the world’s first commercial nuclear power station, and here, energy was generated from 1956 to 2003. The Sellafield site also contains a range of storage ponds built during the 1950s which were intended to support the production of weapon-grade plutonium, and more recently fuels from the United Kingdom’s fleet of nuclear power stations (Reddy et al., 2012; WNA, 2018b). The legacy of activities have left a complex range of nuclear operations at Sellafield, including the decommissioning of redundant facilities associated with the site’s early defense work, and spent fuel management including Magnox and Oxide fuel reprocessing (Gov UK, 2018).

Prior to reprocessing, all irradiated fuel delivered to Sellafield is stored for a period of at least 100 days in water-filled reinforced concrete ponds that allow the decay of short-lived radioisotopes. During storage, the degree of corrosion experienced by the fuel is monitored to determine storage life and optimize water chemistry (Shaw, 1990). The temperature within the ponds is controlled by refrigerant chillers to further limit fuel corrosion, while the levels of both radioactive and non-radioactive ions in the pond waters are controlled by purging cycles of demineralised water adjusted to pH 11.1–11.6 with the addition of sodium hydroxide (Howden, 1987). The main pre-reprocessing storage pond at the Sellafield site is the indoor alkaline storage pond (INP), a concrete wall pond filled with demineralised water, responsible for receiving, storing and mechanically processing spent nuclear fuel (SNF). The SNF, defined as nuclear reactor fuel that has been used to the extent that it can no longer effectively sustain a chain reaction, is received and handled in Sellafield from Magnox and Advanced Gas-cooled Reactor (AGR) stations from across the United Kingdom (Sellafield Ltd, 2015).

Although Sellafield’s nuclear facilities, including the INP, are considered to be oligotrophic with high background levels of radiation, microorganisms have been shown to colonize these inhospitable environments (MeGraw et al., 2018). The presence of diverse microbial communities may impact on site operations and fuel stability. Microorganisms can also play a significant role in the transformations of radionuclides in the environment by altering their chemical speciation, solubility and sorption properties, ultimately impacting their environmental mobility and bioavailability (Lloyd and Renshaw, 2005; Francis, 2012; Newsome et al., 2014a; MeGraw et al., 2018). For example, the interactions between microbial populations and soluble radionuclides in groundwater can lead to precipitation reactions [e.g., via U (VI) or Tc (VII) bioreduction] and subsequent bioremediation (Newsome et al., 2014b). Of particular note within these pond environments is the fate of ^90^Sr and ^137^Cs, which dominate the radionuclide inventory in the water columns in storage ponds at Sellafield (Lang et al., 2019). Previous studies showed that seasonal blooms dominated by the alga *Haematococcus*, have adapted to survive in a circumneutral pH outdoor spent fuel storage pond at Sellafield, and are able to accumulate high levels of these radionuclides (MeGraw et al., 2018).

The accumulation of radionuclides by microbial cells can be driven by a range of processes including biosorption, biomineralization and bioprecipitation (Gadd, 2009), although these are poorly defined in nuclear storage ponds. Biosorption is species-specific and is affected by the chemistry and the pH of the solution, the physiological state of the cells, the cell wall architecture, and the presence of extracellular polymeric substances (EPS) (Comte et al., 2008; Merroun and Selenska-Pobell, 2008). The EPS is especially important, being mainly composed of polysaccharides, proteins, humic substances, uronic acids, nucleic acids, and lipids (Wingender et al., 1999), and containing ionisable functional groups that represent potential binding sites for the sequestration of metal ions (Brown and Lester, 1982; Lawson et al., 1984). Biosorption of divalent cations such as Sr^2+^ is well known (White and Gadd, 1990; Gadd, 2009; Liu et al., 2014), and would be favored in high pH pond systems (Ghorbanzadeh and Mohammad, 2009). Monovalent cations such as Cs^+^ would sorb less strongly than divalent cations (Andrès et al., 2001), although can bioaccumulate in biomass being transported into microbial cells, such as *Rhodococcus*, via potassium transport systems (Tomioka et al., 1992; Avery, 1995a,b). Recent work on a legacy high pH outdoor storage system at Sellafield, identified a *Pseudanabaena* species as the dominant photosynthetic microorganism (Foster et al., 2020a), and lab-based experiments on a culture dominated by a close relative showed increased polysaccharide production following irradiation treatments (Foster et al., 2020b). The polysaccharide production can promote the EPS formation which eventually can impact on ^90^Sr sorption-desorption behavior at alkaline environmental conditions under pond water conditions (Ashworth et al., 2018; MeGraw et al., 2018; Foster et al., 2020b).

Biomineralization reactions can also be linked to radionuclide fate (reviewed by Lloyd and Macaskie, 2000), due to local redox changes (e.g., bioreduction of actinides or key fission products) (Lloyd, 2003), localized alkalinisation at the cell surface (Van Roy et al., 1997) or the accumulation of microbially generated ligands (e.g., phosphate, sulfide, oxalate or carbonate) (White and Gadd, 1990; Macaskie et al., 1992; White et al., 1998; Boswell et al., 2001; Lloyd and Macaskie, 2002). For the latter, induced or mediated carbonate mineralization (MICP) (Braissant et al., 2002), can affect the mobility and sequestration of radionuclides in the near-surface environment (Ferris et al., 1994; Reeder et al., 2001), and has been studied widely due to its importance in the remediation on contaminated Sr systems (Mortensen et al., 2011). Additionally, a variety of microorganisms are able to drive MICP via urea hydrolysis (Fujita et al., 2004; Achal et al., 2012; Bhaduri et al., 2016) or via photosynthetic processes (Ferris et al., 1994; Dittrich et al., 2003; Lee et al., 2014; Zhu and Dittrich, 2016).

Finally microorganisms can affect the physical chemistry of the water-fuel interactions, leading to microbial-influenced corrosion (MIC) and hence fuel material degradation and radionuclide release (Shaw, 1990; Springell et al., 2014; Rajala et al., 2017). In open storage systems, the proliferation of microorganisms (together with the accumulation of radioactive sludge as a result of corrosion in spent fuel ponds) can also adversely impact on pond visibility, increasing the costs of fuel storage, hampering decommissioning operations and also increasing the exposure time to personnel (Wolfram et al., 1996; Jackson et al., 2014).

Recent publications have shown the presence of wide diversity of microorganisms living in SNF ponds, mainly bacteria, photosynthetic cyanobacteria and eukaryotic algae (Chicote et al., 2004, 2005; Sarró et al., 2005; Sellafield Ltd, 2011; Tišáková et al., 2013; Karley et al., 2018; MeGraw et al., 2018; Pipíška et al., 2018; Silva et al., 2018). The observed adaptation mechanisms include biofilm formation (Santo Domingo et al., 1998; Sarró et al., 2005; Bruhn et al., 2009), interactions with radionuclides via biosorption (Tomioka et al., 1992; Adam and Garnier-Laplace, 2003; Ghorbanzadeh and Mohammad, 2009; Dekker et al., 2014) and bioprecipitation (Ferris et al., 1994; Achal et al., 2012; Bhaduri et al., 2016; Zhu and Dittrich, 2016; Bagwell et al., 2018). To date, most published work on the Sellafield site has been on legacy outdoor pond systems (MeGraw et al., 2018; Foster et al., 2020a,b) which are open to external energy sources (including daylight, supporting photosynthetic primary colonizers). Indoor pond systems on the Sellafield site, with lower light intensities, and reduced inputs from atmospheric deposition, have not been studied in such detail.

The aim of this study was to characterize microbial communities of an indoor alkaline spent fuel storage pond (INP) on the Sellafield site, to help understand the microbial ecology of this facility, and the potential forms of metabolism that could underpin colonization. An additional goal was to provide baseline microbial community data, so that the impact of receiving new fuels and stored waste material during upcoming and extensive site-wide decommissioning activities across the Sellafield site can be assessed. The findings of this 30-month survey are discussed in relation to microbial survival to extreme environments (including potential energy sources) and how the extant microbiomes may potentially impact pond management. Microbial communities in the feeding tank supplying the pond system were identified and compared to those in the main and subponds containing spent fuel, to determine which organisms were uniquely adapted to the extreme pond chemistry (e.g., high pH) and high background radiation levels. Throughout the sampling campaign, the presence of hydrogen-oxidizing bacteria (affiliated with the genus *Hydrogenophaga*) in the INP, was consistent with the existence of hydrogen-oxidizing ecosystem, potentially linked to radiolysis in the fuel storage pond.

## Materials and Methods

### Indoor Nuclear Fuel Storage Pond

**FIGURE 1:**
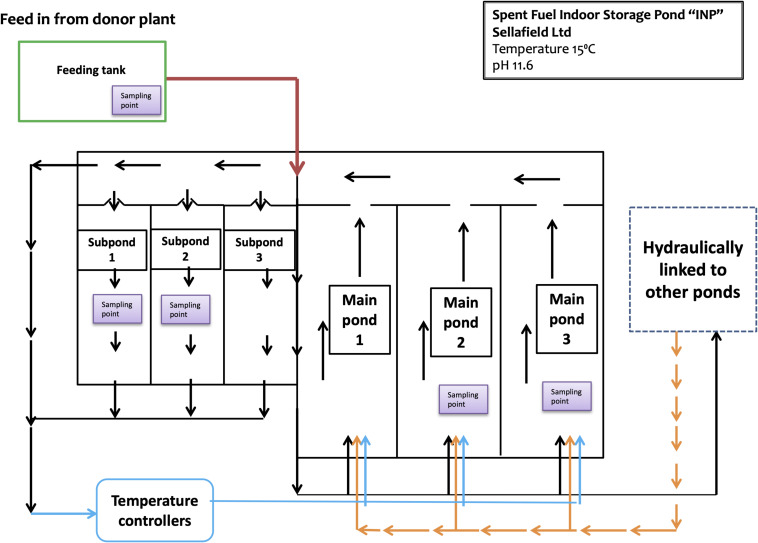
Diagram of the spent fuel indoor storage pond “INP.” It consists of 3 main ponds and 3 subponds linked by a transfer channel which enables water flow. The sampling points are located at the main ponds 2 and 3; subponds 1 and 2; and the head feeding tank (at the top of the pond).

The INP is an indoor pond complex divided into three main ponds and three subponds linked by a transfer channel that enables water flow (Figure 1). In order to control the pond-water activity and quality, there is a continuous “once through” purge flow; pond-water from the main ponds flows into the transfer channel and enters the recirculation pump chamber where it is continuously pumped round a closed circulation loop and through a heat exchanger system, which cools the pond-water before it is recycled into the main ponds. Through the control feed, purge and re-circulation flow rates, the water depth is maintained at 7 ± 0.05 m. The purge flow can be either from a donor plant or from other hydraulically linked ponds within the Sellafield complex. The temperature and pH are controlled at 15°C and 11.6, respectively. Analyzed samples were taken from three designated main areas: main ponds (MP2 and MP3), subponds (SP1 and SP2), and from the Feeding tank (FT) of the donor plant, where the demineralised water used to feed the INP is stored.

### Samples

Thirty samples were taken from three designated areas resulting on five sampling points (Table 1) for a period of 30 months (October 2016–April 2019).

**TABLE 1:** Distribution of samples taken for a period of 30 months from different areas within the SNF pond, and analyzed using high-throughput (Illumina) DNA microbial profiling.

**Main sampling areas**	**Feeding tank (FT)**		**Main ponds (MP)**				**Subponds (SP)**			
**Sampling points**	**Feeding tank (FT01)**	**Number of sequences (after QC)**	**Main pond 2 (MP2)**	**Number of sequences (after QC)**	**Main pond 3 (MP3)**	**Number of sequences (after QC)**	**Subpond 1 (SP1)**	**Number of sequences (after QC)**	**Subpond 2 (SP2)**	**Number of sequences (after QC)**
**Date \**										
October 2016	FT01 FT02	183,179 153,474	MP2_01	171,186	MP3_01	147,661	–		–	–
January 2017	–	–	–	–	–	–	SP1_01*	NA	SP2_01*	NA
June 2017	–	–	MP2_02	180,440	MP3_02	170,531	–	–	–	–
October 2017	–	–	MP2_03	110,308	MP3_03	151,014	–	–	–	–
January 2018	–	–	MP2_04	209,292	MP3_04	174,953	SP1_02	178,489	SP2_02	155,145
June 2018	–	–	MP2_05	173,003	MP3_05	163,231	SP1_03	139,082	SP2_03	175,436
November 2018	–	–	MP2_06	138,631	MP3_06	146,546	SP1_04	175,585	SP2_04	133,749
February 2019	–	–	MP2_07	156,500	MP3_07	124,047	SP1_05	150,385	SP2_05	109,585
April 2019	–	–	MP2_08	134,663	MP3_08	148,415	SP1_06	93,102	SP2_06	133,452

Samples SP1_01 and SP2_01 (*) were not sequenced using the Illumina platform but instead were analyzed using culturing techniques (with Sanger sequencing of isolated pure cultures).

Water samples from the FT were considered non-active and were shipped directly to the University of Manchester in October 2016 and stored in the dark at 10°C. Water samples from the MP 2 and 3 and SP 1 and 2 were radioactive, hence appropriate handling procedures were required. The protocols for these samples were developed and applied under Command and Control regimes by Sellafield Ltd. and NNL, with samples transferred directly from the pond to the NNL Central Laboratory (National Nuclear Laboratory, Cumbria, United Kingdom), where DNA was extracted and the samples were checked for radioactivity in line with the Environmental Permits and Nuclear Site licenses held by Sellafield Ltd. Extracted DNA samples free from significant radionuclide contamination were shipped to the University of Manchester and stored in the freezer (−20°C) until use.

In addition to microbial profiling via DNA analyses, a complementary “cultivation-dependent” approach was also adopted to help further characterize the pond microbial community composition. Two low-volume samples (approximately 5 mL) from subponds 1 and 2 (Figure 1) were analyzed by traditional culturing techniques. The subponds are more radioactive than the main ponds, but the temperature and pH values are maintained at the same values as the main ponds, 21°C and 11.6, respectively. The typical pond water activities are 1,000 Bq/ml β (Table 2).

**TABLE 2:** Parameters measured in the indoor alkaline spent fuel storage pond (INP).

**Parameter**	**Feeding tank (FT)**	**Main ponds (MP)**	**Subponds (SP)**
pH	11.5124 ± 0.01	11.5779 ± 0.01	11.6 ± 0.02
Temperature (°C)	14.1105 ± 0.04	14.4607 ± 0.04	14.168 ± 0.037
Na^+^ (μg/mL)	80.4114 ± 0.41	81.1753 ± 0.35	81.2830 ± 0.38
TOC (μg/mL)	2.11 ± 0.24	2.07 ± 0.23	2.02 ± 0.22
Phosphates PO_4_^–2^ (g/mL)	0.00	0.00	0.00
Nitrates NO_3_^–2^ (μg/mL)	0.01 ± 0.01	0.01 ± 0.01	0.01 ± 0.01
Beta AC (Bq/mL)	NA	1,131.6470 ± 28.88	1,117.44853 ± 30.77

*Data provided by Sellafield Ltd. Mean ± standard error is presented for each parameter. NA, not available data.*

### Cultivation-Independent DNA Analyses of Microbial Communities

#### DNA Extraction and PCR Amplifications

The MoBio PowerWater DNA isolation kit (MoBio Laboratories, Inc., Carlsbad, CA, United States), was used to extract DNA from water samples of approximately 1 L. The DNA was eluted to a final volume of 100 μL, and stored at 4°C until they were transported to UoM, where it was kept at −20°C to await further analyses.

Polymerase Chain Reaction (PCR) amplification was performed from the extracted DNA using a Techne Thermocycler (Cole-Parmer, Staffordshire, United Kingdom). Primers used for detection of bacterial 16S rRNA gene amplification were the broad-specificity 8F forward primer and the reverse primer 1492R (Eden et al., 1991). The primers used to detect eukaryotic organisms, targeting the 18S rRNA gene, were Euk F forward primer and the reverse primer Euk R (DeLong, 1992) whilst the archaeal primers that targeted the 16S rRNA gene, were forward primer 21F and reverse primer 958R (DeLong, 1992). The PCR reaction mixtures contained; 5 μL 10× PCR buffer, 4 μL 10 mM dNTP solution (2.5 mM each nucleotide), 1 μL of 25 μM forward primer, 1 μL of 25 μM reverse primer, and 0.3 μL Ex Takara Taq DNA Polymerase. The final volume was made up to 50 μL with PCR grade water, which included the addition of 2 μL of sample. The thermal cycling protocol used was as follows for the bacterial 8F and 1492R primers; initial denaturation at 94°C for 4 min, melting at 94°C for 30 s, annealing at 55°C for 30 s, extension at 72°C for 1 min (35 cycles with a final extension at 72°C for 5 min) (Eden et al., 1991). For the eukaryotic 18S rRNA gene amplification, the temperature cycle was; initial denaturation at 94°C for 2 min, melting at 94°C for 30 s, annealing at 55°C for 1.5 min, extension at 72°C for 1.5 min for a total of 30 cycles, and final extension at 72°C for 5 min (DeLong, 1992). For archaeal 16S rRNA genes the thermal cycle protocol consisted of an initial denaturation step at 94°C for 4 min, melting at 94°C for 45 s, annealing at 55°C for 30 s, extension at 72°C for 1 min (for a total of 30 cycles) and a final extension step at 72°C for 5 min (DeLong, 1992).

The purity of the amplified PCR products were checked by electrophoresis using a 1% (w/v) agarose gel in 1X TAE buffer (Tris-acetic acid-EDTA). DNA was stained with SYBER safe DNA gel stain (Thermofisher), and then viewed under short-wave UV light using a BioRad Geldoc 2000 system (BioRad, Hemel Hempstead, Herts, United Kingdom).

#### Quantitative Polymerase Chain Reaction

Quantitative Polymerase Chain Reaction (qPCR) of the prokaryotic 16S rRNA gene was performed by using Brilliant II Sybr Green qPCR Master Mix and the MX3000P qPCR System (Agilent Genomics, Headquarters, Santa Clara, CA, United States). The qPCR master mix contained 0.4 μL 8F forward primer (25 μM), and 0.4 μL 519R reverse primer (25 μM) (Turner et al., 1999), 0.4 μL of one in five diluted Rox reference dye, 12.5 μL of 2x qPCR Sybr green master mix, and Roche PCR Grade water to make up a final volume of 23 μL. Finally, 2 μL of sample was added. A standard curve from known serial dilutions of template DNA was constructed by plotting the CT (cycle threshold) values to verify the presence of a single gene-specific peak and the absence of primer dimer. The cycling conditions consisted of one cycle of denaturation at 94°C for 10 min, followed by 35 three-segment cycles of amplification (94°C for 30 s, 50°C for 30 s and 72°C for 45 s). Fluorescence was automatically measured during the PCR amplification, and one three-segment cycle of product melting (94°C for 10 min, 50°C for 30 s and 94°C for 30 s). Gene quantification was achieved by determining the threshold cycle (CT) of the unknown samples compared to the standard curve. The baseline adjustment method for the Mx3000 (Agilent) software was used to determine the Ct in each reaction. All samples were amplified in triplicate, and the mean was used for further analysis. In order to quantify the concentration of target genes, the absolute quantification by the standard-curve (SC) method was used (Brankatschk et al., 2012). To determine the abundance of cells mL^–1^ of sample, the total number of 16S rRNA genes determined by qPCR was adjusted to the approximated number of 16S rRNA gene copy numbers reported for members of the Proteobacteria; specifically for classes alpha and beta the average number of copies is reported to be 4 (Větrovský and Baldrian, 2013). A paired-samples (one-tailed) *t*-test (Sullivan, 2017) was conducted to compare the number of DNA copies over time in the MP and SP areas). Analysis was carried out on MP samples that were collected between 2016 and 2019, whilst the SP samples were collected between 2018 and 2019.

#### DNA Sequencing

Sequencing of 16S rRNA gene PCR amplicons was conducted using the Illumina MiSeq platform (Illumina, San Diego, CA, United States) targeting the V4 hyper variable region (forward primer, 515F, 5′-GTGYCAGCMGCCGCGGTAA-3′; reverse primer, 806R, 5′-GGACTACHVGGGTWTCTAAT-3′) for 2 × 250-bp paired-end sequencing (Illumina, San Diego, CA, United States) (Caporaso et al., 2011, 2012). PCR amplification was performed using the Roche FastStart High Fidelity PCR System (Roche Diagnostics Ltd., Burgess Hill, United Kingdom) in 50 μL reactions under the following conditions; initial denaturation at 95°C for 2 min, followed by 36 cycles of 95°C for 30 s, 55°C for 30 s, 72°C for 1 min, and a final extension step of 5 min at 72°C. The PCR products were purified and normalized to ∼20 ng each using the SequalPrep Normalization Kit (Fisher Scientific, Loughborough, United Kingdom). The PCR amplicons from all samples were pooled in equimolar ratios. The run was performed using a 4 pM sample library spiked with 4 pM PhiX to a final concentration of 10% following the method of Schloss and Kozich (Kozich et al., 2013).

Raw sequences were divided into samples by barcodes index I5 and I7 (up to one mismatch was permitted) using a sequencing pipeline. Quality control and trimming (Q score of 20, and a minimum length of 250 base pairs) was performed using Cutadapt (Martin, 2011), FastQC (Bioinformatics, 2018), and Sickle (Joshi and Fass, 2011). MiSeq error correction was performed using SPADes (Nurk et al., 2013). Forward and reverse reads were incorporated into full-length sequences with Pandaseq (Masella et al., 2012). Chimeras were removed using ChimeraSlayer (Edgar et al., 2011), and OTU’s were generated with UPARSE (Edgar, 2013). OTUs were classified by Usearch (Edgar, 2010) at the 97% similarity level, and singletons were removed. Rarefaction analysis was conducted using the original detected OTUs in Qiime (Caporaso et al., 2010). The taxonomic assignment was performed by the RDP classifier (Wang et al., 2007). OTU sequences were submitted to the NCBI GenBank repository under the Bioproject number PRJNA660452, detailed accession numbers are indicated in Supplementary Table 2.

### Culturing and Identification of the Pond Microorganisms

A complementary culture-dependent approach was used to help characterize the microorganisms present. To facilitate this, a series of 10-fold dilutions of water samples from the subponds 1 and 2 were plated onto fresh solid media. A range of complex or semi-defined solid media (at 10, 50, and 100% concentrations) were used (Supplementary Table 3) including Luria Bertani (LB) (Sezonov et al., 2007), Nutrient Agar (NA) (Misal et al., 2013), and Minimum medium DL (Lovley et al., 1984) at a range of pH values (7, 10, and 11). The marine medium of Zobell was also selected for isolation of alpha and gammaproteobacteria (Brettar et al., 2004) that had been detected in the pond using cultivation-independent DNA sequencing. Finally the fully defined minimal medium M9 (Neidhart et al., 1974) was also used at a range of concentrations and pH. The M9 medium contained no added carbon, selecting for autotrophic oligotrophs.

The isolated colonies were then resuspended in 10 mL of fresh liquid media and grown aerobically for 48 h. Cells were harvested by centrifuging at 3,500 *g* for 10 min, and supernatant was removed leaving the cell pellet and 100 μL of culture medium. DNA was extracted separately from the cell pellets using the Power Biofilm DNA Isolation Kit (MoBio Laboratories, Inc., Carlsbad, CA, United States). The DNA was eluted to a final volume of 100 μL, and stored at 4°C until use.

The 16S rRNA gene sequences of the isolates were determined by the chain termination sequencing method to facilitate phylogenetic analyses of the pure cultures (Slatko et al., 1999). PCR amplification was performed from the extracted DNA using a Techne Thermocycler (Cole-Parmer, Staffordshire, United Kingdom). Two PCR mixtures were prepared (one for each primer) and contained 3.5 μL 5X PCR buffer, 0.15 μL of 25 μM primer, and 1 μL Terminator BigDye (Thermo Fisher Scientific, Waltham, MA, United States), 1 μL of DNA was added to each tube, and was made to a final volume of 15 μL with PCR grade water. The thermal cycling protocol used was adapted for the primers as follows; initial denaturation at 96°C for 6 min, melting at 94°C for 40 s, annealing at 55°C for 15 s, extension at 60°C for 3 min; 30 cycles, and a final extension at 60°C for 5 min (Lorenz, 2012). The resulting PCR products were purified using the GlycoBlue coprecipitant protocol AM9516 (Thermo Fisher Scientific, Waltham, MA, United States), and the resulting pellets were then sequenced. An ABI Prism BigDye Terminator Cycle Sequencing Kit was used in combination with an ABI Prism 3730XL Capillary DNA Analyzer (Applied Biosystems, Warrington, United Kingdom). The primers 8F and 1592R were used for initial amplification and sequencing: 8F 5′-AGA GTT TGATCC TGG CTC AG-3′, and 1492R 5′-TAC GGY TAC CTT GTTACG ACT T-3′ (Lane et al., 1986). Sequences (typically 950 base pairs in length) were aligned with Muscle (Edgar, 2004) via the MEGA software (Kumar et al., 2016) version X (Kumar et al., 2018), then sequences were compared against the GenBank NCBI database using the BLAST program packages and matched to the most similar known 16S rRNA gene sequences (affiliations are detailed on Supplementary Figure 1).

## Results

The aim of this study was to characterize the microbial populations living under the inhospitable high pH, oligotrophic and high background radiation conditions within an INP at the Sellafield complex. Duplicate samples of the purge waters of the FT were collected in October 2016. This non-radioactive purge water feeds into the INP, and therefore the analysis set out to determine the microbial community present in these samples that could seed the INP. We received an initial sample from the SP (in duplicate) in which the sole purpose was try to get the microorganisms present in the pond into culture. We were then provided with further samples (January 2018 onward) from this region of the INP facility to carry out next generation sequencing on. Since culture dependent techniques do not reveal the whole microbial community, it was important to use DNA sequencing techniques to better understand what microorganisms inhabit the SP.

### Cell Density

**FIGURE 2:**
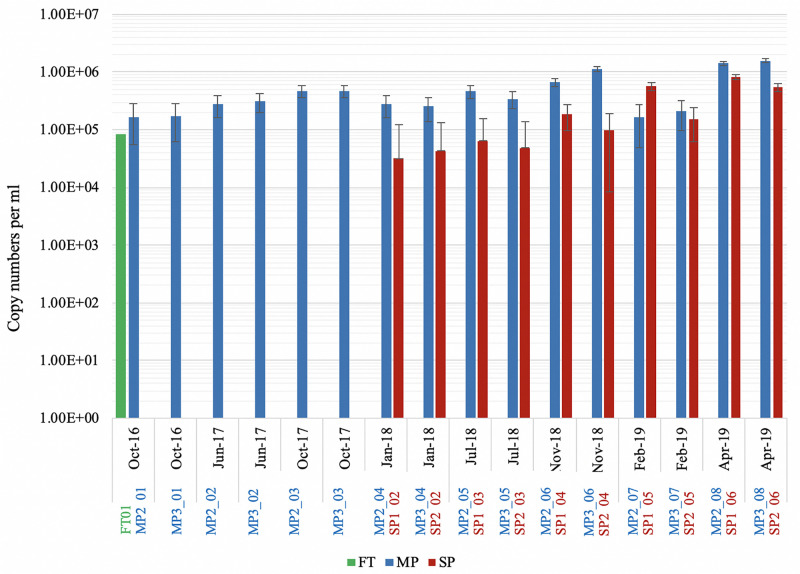
QPCR results show the average number of copies per mL per sampling site and time (FT: feeding tank, MP: main ponds and SP: subponds). Copy numbers are expressed on log scale and error bars represent standard error. Missing bars represent lack of samples for certain sampling dates (FT and SP). A standard curve for QPCR reaction was calculated at concentration ranging from 7.53 × 10^−4^ to 7.530 × 10^3^ ng mL^−1^ (2.8 × 10^3^−2.8×10^12^ DNA copies) to estimate the concentration of DNA in the samples.

In order to get an estimate of the overall cell density of the microorganisms that inhabit this facility, qPCR was carried out. Only the 16S rRNA gene could be detected in the extracted DNA samples, PCR using the eukaryotic 18S rRNA and the archaeal specific 16S rRNA primers did not result in any detectable amplification. Copy numbers were lower in the FT (μ = 8.1 × 10^4^, SE = 3.9 × 10^4^ DNA copies) and SP (μ = 2.5 × 10^5^, SE = 8.8 × 10^4^ DNA copies), while MP values ranged from 2.5 × 10^5^ to 1.5 × 10^6^ DNA copies (Figure 2), peaking in MP2_08 and MP3_08 (1.4 × 10^6^ and 1.56 × 10^6^ DNA copies, respectively; April 2019) and in MP3_06 (1.1 × 10^6^ DNA copies, November 2018). The results showed that the number of DNA copies in the MP from the period 2016–2017 (μ = 2.9 × 10^5^, SD = 1.1 × 10^5^) to the period 2018–2019 (μ = 1.4 × 10^5^, SD = 5.5 × 10^5^) increased significantly [*t*(16) = 2.09, *p* < 0.05]. Likewise, the results on the SP showed that the number of DNA copies from 2018 (μ = 7.8 × 10^4^, SD = 5.7 × 10^4^) to 2019 (μ = 7.8 × 10^5^, SD = 2.7 × 10^5^) increased significantly [*t*(10) = 2.71, *p* < 0.05] (detailed statistical data is provided in Supplementary Table 1).

### Identification of Microorganisms by Next Generation DNA Sequencing

**FIGURE 3:**
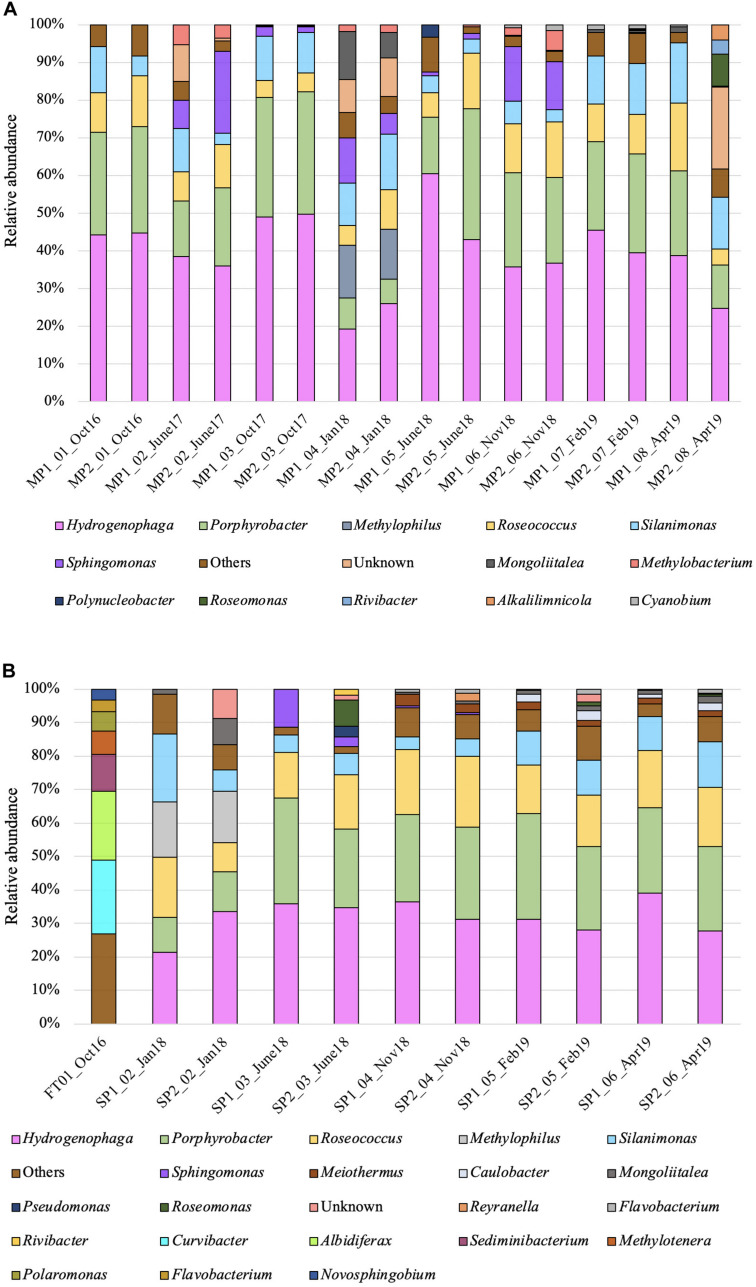
Phylogenetic affiliations (closest known genera) of microorganisms detected in the indoor nuclear pond (INP) at Sellafield: **(A)** microbial community in the main pond (MP); **(B)** microbial community in the feeding tank (FT) and subponds (SP), based on Illumina sequencing of the prokaryote 16S rRNA gene. Only genera that contained more than 1.5% of the total number of sequences are shown.

Over a 30-month sampling campaign a total of 30 samples (Table 1) from three sampling areas (FT, MP, and SP) were analyzed by 16S rRNA gene sequencing on the Illumina MiSeq. The initial sampling (by duplicate) was taken from the feeding head tank (FT) supplying the pond complex with demineralised water adjusted to pH 11.6 in October 2016, to help identify organisms present in the background waters, and hence (by comparison) help identify the organisms that were exclusively present in the INP main and subponds (Figure 3).

DNA extracted from the pond samples were assessed using PCR with 3 primer sets to screen for the presence of the prokaryotic and archaeal 16S rRNA genes and the eukaryotic 18S rRNA gene. However, only prokaryotic 16S rRNA gene amplification products were detected, and it was therefore concluded that eukaryotic and archaeal microorganisms were absent, or below the limit of detection. The 16S rRNA gene was targeted for sequencing using the Illumina MiSeq next generation sequencing platform, and analyzed using a bespoke bioinformatics platform which included comparison to prokaryotic gene sequences deposited in the NCBI databases.

Samples from the main ponds (MP) were dominated [mean ± standard deviation (SD)] by Proteobacteria (92.68 ± 6.26%) and Bacteroidetes (5.25 ± 6.93%). Organisms affiliated with the phylum Cyanobacteria were not detected in the initial samples, but were detected at subsequent times (from October 2017 to April 2019), although at a relative abundance of less than 3%. Samples from the subponds (SP) were also dominated (mean ± SD) by Proteobacteria (90.33 ± 4.01%) and Bacteroidetes (3.67 ± 2.19%), while the relative abundance of Cyanobacteria was again low (0.88 ± 0.52%). In addition, other phyla were detected at higher levels at specific dates in the subponds including organisms affiliated with the Actinobacteria (6.96 ± 2.13%, January 2018), Armatimonadetes (5.54 ± 1.78%, June 2018) and Deinococcus-Thermus groups (3.5 ± 0.95% from November 2018). Samples from the feeding tank (FT) were also dominated (mean ± SD) by Proteobacteria (73.3 ± 3.36%), Bacteroidetes (16.62 ± 3.6%) and Actinobacteria (2.58 ± 1.23%) (Supplementary Figure 2).

At the genus level the FT was dominated (mean ± SD) by close relatives of *Curvibacter* (21.4 ± 1.33%, Betaproteobacteria, 1 OTU), *Rhodoferax* (19.97 ± 0.51%, Betaproteobacteria, 1 OTU), *Sediminibacterium* (10.99 ± 0.85 %, Bacteroidetes, 2 OTUs), *Polaromonas* (5.51 ± 5.74%, Betaproteobacteria, 2 OTUs), *Methylotenera* (6.74 ± 6.29%, Betaproteobacteria, 2 OTUs), *Novosphingobium* (3.26 ± 1.87%, Alphaproteobacteria, 2 OTUs), *Flavobacterium* (3.40 ± 3.33%, Bacteroidetes, 2 OTUs), *Unidibacterium* (3.11 ± 0.52%, Betaproteobacteria, 2 OTUs). Approximately 26.25 ± 1.33% of the total OTUs (26) represented unidentified organisms.

Microbial distributions were consistent at all sampling times within the main ponds (MP) (Figure 3A). Overall at the genus level the microbial diversity was dominated (mean ± SD) by 1 OTU belonging to genus *Hydrogenophaga* (Betaproteobacteria), representing up to 38.76 ± 10.03% of the total population. The remaining community consisted of *Porphyrobacter* (21.57 ± 8.44%, Alphaproteobacteria, 1 OTU), *Roseococcus* (9.82 ± 4.15%, Alphaproteobacteria, 3 OTUs), *Silanimonas* (9.49 ± 4.50%, Gammaproteobacteria, 1 OTU), *Sphingomonas* (7.84 ± 6.82%, Alphaproteobacteria, 2 OTUs), and *Synechococcus* (0.91 ± 0.49%, Cyanophyceae, 3 OTUs). The exception was one set of samples taken on January 2018 (MP2_04 and MP3_04), where broad microbial diversity was recorded and the abundance of *Hydrogenophaga*, and *Porphyrobacter* dropped to 22.52 ± 4.73% and 7.40 ± 1.19%, respectively. Additionally, representatives of the genera *Methylophilus* (13.61 ± 0.69%, Betaproteobacteria, 1 OTU) and *Mongoliitalea* (9.80 ± 4.37%, Bacteroidetes, 2 OTUs) were exclusively identified at this sampling time. Unidentified (uncultured) sequences, although detected at all sampling times, represented more than 2% of the total community in samples MP2_02 (June 2017, 8.84 ± 6.77%, 24 OTUs), MP2_04 and MP3_04 (January 2018, 8.67 ± 1.08% and 10.19 ± 6.77%, 38 OTUs) and MP3_08 (April 2019, 21.48 ± 6.77%, 47 OTUs).

The microbial profiles of the subponds (SP) were similar to the main ponds (MP), and were dominated (mean ± SD) by representatives of the genera *Hydrogenophaga* (30.55 ± 5.73%, Betaproteobacteria, 1 OTU), *Porphyrobacter* (23.06 ± 7.52%, Alphaproteobacteria, 1 OTU), *Roseococcus* (15.44 ± 3.45%, Alphaproteobacteria, 2 OTUs), *Silanimonas* (8.73 ± 4.34%, Gammaproteobacteria, 2 OTUs), and *Sphingomonas* (2.4 ± 4.21%, Alphaproteobacteria, 3 OTUs). Samples SP1_02 and SP2_02 (January, 2018) showed a few differences with close relatives affiliated to genus *Methylophilus* (13.61 ± 0.69%, Alphaproteobacteria, 1 OTU) detected in these samples only (Figure 3B).

When viewed at the phylum level all three sampling areas (5 sampling points in total) showed similar profiles, as they were dominated by Proteobacteria, however, when looking at the affiliations at the genus level the microbial communities at each sampling point could be seen to differ substantially. Data would seem to suggest that the microbial community compositions in the MP, SP and FT samples represent distinct ecosystems, most likely linked to the impacts of the spent fuel in the INP environment.

### Cultivation-Dependent Analysis for Determining Microbial Diversity in the INP

After 7 days of incubation, growth was detected exclusively in the undiluted samples (100) from plates containing non-defined complex media (DL, NA, and Zobell media; Supplementary Table 3). CFU mL^–1^ were between 700 and 1,000 mL^–1^ for each medium and eleven distinct colony morphologies were noted. Representative single colonies were isolated and identified by sequencing using the dideoxynucleotide technique. The presence of colonies was not detected on the fully defined media (minimal media M9).

Overall, representatives of four different genera were identified by 16S rRNA gene sequencing. Representatives most closely related to species of the genus *Algoriphagus* (isolates S01, 91.5% similarity; S05, 91% similarity; S06, 91.5% similarity; and S07, 89.5% similarity) were isolated on DL and NA agars, and produced light pink-colored, rod-shaped and raised colonies (1–2 mm diameter). Organisms most closely related to members of *Echinicola* genus (isolates S02, 91% similarity; S08, 88% similarity; and S09, 93.5% similarity) were obtained on the DL and NA agar plates, and produced red-colored colonies, that were rod-shaped with raised elevation (2–3 mm diameter). Strains S03, S10, and S11 were isolated from DL, NA and Zobell plates; were rod-shaped, translucent and had raised colonies (2–3 mm diameter) and were affiliated to an unclassified genus from the family Cyclobacteriaceae (S03, 93.5% similarity; S10, 85% similarity and S11, 91% similarity). Finally, a close relative to genus *Bacteroides* (strain S04, 91.5% similarity) was isolated from the DL plates and produced short round-shaped, bright-orange raised colonies (1–2 mm diameter). All eleven isolated strains belonged to the phylum Bacteroidetes (specific details on similarity and media are shown on Supplementary Table 4).

Members belonging to genus *Echinicola* (phylum Bacteroidetes) were previously detected in the MP and SP samples by DNA-based techniques; however, they did not represent a major component of the community. More precisely, members of the genus *Echinicola* were detected in samples MP2_03 and MP3_03 (October 2017) at a relative abundance of 0.28 and 0.39%, respectively (Supplementary Table 5).

## Discussion

The present research was focused on characterizing the microbial community of a Sellafield INP complex containing main ponds (MP), subponds (SP) and a feeding head tank (FT) over a period of 30 months. The results showed that bacteria affiliated with a range of phylogenetic groups are able to survive and colonize the different areas across the INP complex.

Microbial diversity within the FT, an oligotrophic and hyper-alkaline environment, was dominated by members belonging to the Proteobacteria and Bacteroidetes. Previous studies showed that oligotrophic conditions do not prevent microbial colonization and allow microbial communities to display diverse adaptation mechanisms (Kawai et al., 2002; Kulakov et al., 2002; Chen et al., 2004). Specifically, organisms associated to Proteobacteria and Bacteroidetes have been identified previously in similar oligotrophic environments, including industrial ultrapure water (Galès et al., 2004; Bohus et al., 2011; Proctor et al., 2015). Microbial colonization in such environments has been linked to low levels of residual organic matter in the system, originating from dead microbial cells that and to biofilm formation on the walls, linked to planktonic cells delivered by water recirculation in the pond areas (Bohus et al., 2011). Organisms detected in the FT area are reported to support diverse forms of heterotrophic metabolism, which could occur within the FT. For example, members of the genera *Rhodoferax* (Finneran et al., 2003; Risso et al., 2009), *Curvibacter* and *Sediminibacterium* (Qu and Yuan, 2008; Ding and Yokota, 2010; Kang et al., 2013, 2014; Ma et al., 2016) are able to oxidize a range of complex organic compounds, while *Methylotenera* can utilise reduced one-carbon compounds (methylotrophy) such as methanol as energy sources (Kalyuzhnaya et al., 2006, 2011). However, the source of carbon and the source of energy microorganisms use in the FT remains to be investigated.

Although the INP has a continuous pond purge, the main ponds (MP) and subponds (SP) contained stable microbial populations with similar community profiles, which contrasted with the distinct microbiome of the FT. Key organisms detected in MP and SP samples included species of *Hydrogenophaga*, *Silanimonas*, *Porphyrobacter*, and *Roseococcus*.

In addition to the oligotrophic and hyper-alkaline characteristics of the MP and SP areas, spent nuclear fuel results in high background radioactivity, which further challenges the microbial community in the pond. Despite these adverse conditions, microbial colonization of similar spent fuel storage systems has been documented (Santo Domingo et al., 1998; Galès et al., 2004; Bruhn et al., 2009), and dominated by organisms associated to the phyla Proteobacteria (Chicote et al., 2004; Bagwell et al., 2018; MeGraw et al., 2018; Silva et al., 2018), Firmicutes (Sarró et al., 2005), Actinobacteria (Sarró et al., 2005), Cyanobacteria (MeGraw et al., 2018; Silva et al., 2018; Foster et al., 2020a), and Deinococcus-Thermus (Masurat et al., 2005). Whilst it was not possible to identify any eukaryotic organisms in the INP, other studies have identified fresh water microalgae (Rivasseau et al., 2016; MeGraw et al., 2018) and Fungi (Chicote et al., 2004; Silva et al., 2018) in both indoor and outdoor facilities. Although the energy sources supporting microbial growth in these systems remains largely uncharacterized, it is possible that radiolysis could play a direct role in supporting microbial growth. The presence of alpha, beta and gamma radiation from the spent fuel can promote the radiolysis of water, driving the formation of short-lived, highly oxidizing free radical species, such as -OH and H_2_O_2_ (Shoesmith, 2000; Jonsson et al., 2007) and also the production of H_2_ (Brodie et al., 2006; Libert et al., 2011) that could be utilized by hydrogen-oxidizing bacteria (Knallgas bacteria) (Yu, 2018). The most abundant organism in the MP and SP areas in this study were affiliated with the genus *Hydrogenophaga* (35.61 ± 9.42%), which comprise aerobic, chemoorganotrophic organisms that use hydrogen as an energy source (Willems et al., 1989; Kampfer et al., 2005; Yoon et al., 2008). Members of genus *Hydrogenophaga* are present in a variety of natural and engineered (e.g., waste water) environments (Lambo and Patel, 2006; Fahy et al., 2008; Yoon et al., 2008; Schwartz et al., 2013), including hyper alkaline sites such as Allas Springs, Cyprus where the pH was 11.9, similar to the alkaline conditions to the INP waters (pH 11.6) (Rizoulis et al., 2016) and serpentinizing springs (pH 11.6, The Cedars, Los Angeles, CA, United States) (Suzuki et al., 2014). The presence of *Hydrogenophaga* as a key microbial component during all the sampling times suggests that the metabolism of H_2_ may be occurring within the pond, which is of particular interest since oxidation of hydrogen could also be linked to the reduction of a range of electron acceptors, including radionuclides (Lloyd, 2003).

Hydrogen metabolism has not been reported for the remaining microbial community identified in the INP. *Porphyrobacter*, an aerobic anoxygenic phototrophic bacteria (AAP), has the ability to harvest energy photosynthetically (Hanada et al., 1997; Yoon et al., 2004; Liu et al., 2017); however, given the limited light availability in the pond, it is unlikely to be photosynthetically active in the INP. Members of this genus have been shown to be well-adapted to life in environments with light restrictions using light energy via Bacteriochlorophyll α synthesized in the dark (Fuerst et al., 1993; Yoon et al., 2008; Liu et al., 2017). Members of the *Roseococcus* genus, are obligate aerobes and chemoorganotrophic, they contain Bacteriochlorophyll α and carotenoid pigments (Boldareva et al., 2009; Yurkov, 2015), and are also able to grow in the dark (Yurkov et al., 1994). *Sphingomonas* species are metabolically versatile and can use a wide range of compounds as energy sources (Lee et al., 2001; Feng et al., 2014; Singh et al., 2015) such as polycyclic aromatic hydrocarbons (Leys et al., 2004); and contains ubiquinone Q-10, a molecule involved in respiratory functions (Niharika et al., 2012) where hydrogen, a potentially abundant energy source in the MP and SP areas, is required. *Roseomonas* species also contain ubiquinone Q-10 (Kim et al., 2009; Wang et al., 2016), and have the ability to grow on biofilms to protect themselves from adverse conditions (Diesendorf et al., 2017), such as those present in this radioactive facility. Microorganisms associated with the oxygenic and phototrophic phylum Cyanbacteria (Peschek, 1999), were much less abundant (identified as genera *Synecochoccus* and *Cyanobium*), which is likely to be a result of the low levels of light in the INP. The metabolic pathways utilized in the pond to facilitate their growth are not known yet and further work is required to better understand this.

Finally, agar-based cultivation approaches were tested alongside DNA-based approaches in this study, and resulted in the isolation of bacteria from the family *Cyclobacteriacea*, but proved unsuccessful for targeting organisms that were numerically dominant within the INP complex. Whilst the isolated organisms do not represent the major components of the pond microbial communities identified by NGS techniques, these new findings showed that organisms affiliated with the genera *Algoriphagus* and *Echinicola* were able to tolerate alkaline conditions (and given the source of inocula, presumably high levels of radioactivity and oligotrophic nutrient conditions), in stark contrast to the neutral pH environments they are normally associated with (Tiago et al., 2004; Yoon et al., 2004; Alegado et al., 2013; Kang et al., 2013; Misal et al., 2013; Glaring et al., 2015).

Overall this study reinforces the view that cultivation-independent molecular ecology techniques are crucial first steps in understanding the microbial dynamics in oligotrophic SNPs, offering the benefits of high-throughput sequencing of DNA that has been purified away from contaminating radionuclides present in the pond waters. This opens up the way for more detailed metagenomic analyses which are ongoing in our laboratories, alongside more targeted research on the impact of extant microbiomes within spent nuclear fuel storage ponds on the speciation and fate of key radionuclides present within the pond systems, and also the integrity of stored fuel materials.

## Data Availability Statement

The datasets presented in this study can be found in online repositories. The names of the repository/repositories and accession number(s) can be found in the article/Supplementary Material.

## Author Contributions

SR-L developed the concept, analyzed and interpreted data, and wrote the manuscript. CB performed the DNA sequencing runs. LF performed the DNA extractions at NNL labs. KM contributed to concept development. NC data curation and provision of samples from the facility and reviewed the manuscript. JL developed the concept and extensively reviewed the manuscript. All the authors read and approved the final manuscript.

## Conflict of Interest

Additional funding was provided by Sellafield. The funder played no role in the study design and analysis, decision to publish, or preparation of the manuscript. Sellafield Ltd., did provide pond samples and detailed pond data was collected as part of routine operations. NC was employed by Sellafield Ltd. The remaining authors declare that the research was conducted in the absence of any commercial or financial relationships that could be construed as a potential conflict of interest.
