# Supplementary material for: Identification of a Stable Hydrogen-Driven Microbiome in a Highly Radioactive Storage Facility on the Sellafield Site
Source: Front Microbiol. 2020 Nov 24;11:587556. doi: 10.3389/fmicb.2020.587556 (PMC7732693; doi:10.3389/fmicb.2020.587556)
Supplement: Supplementary file 1 [file Data_Sheet_1.docx]

Supplementary Material

**Supplementary Table 1**. Paired-samples T-test one-tailed data to compare the qPCR mean values detected on the MP and SP

| **Area** | **Main ponds 2 and 3 (MP2 and MP3)** | | **Subponds 1 and 2 (SP1 and SP2)** | |
| --- | --- | --- | --- | --- |
| Period of analysis | **2016-2017** | **2018-2019** | **2018** | **2019** |
| Mean (μ) | 298,317.527 | 743,943.182 | 78,127.663 | 520,229.116 |
| Standard deviation (σ) | 115,819.377 | 553,866.579 | 57,000.111 | 273,687.257 |
| Statistical test | **Paired-samples T test (one-tailed)** | | | |
| T value | **2.0901** | | **2.7186** | |
| Critical value (T) at α = 0.05 | 1.895 | | 2.353 | |
| p value | 0.03745 | | 0.02653 | |


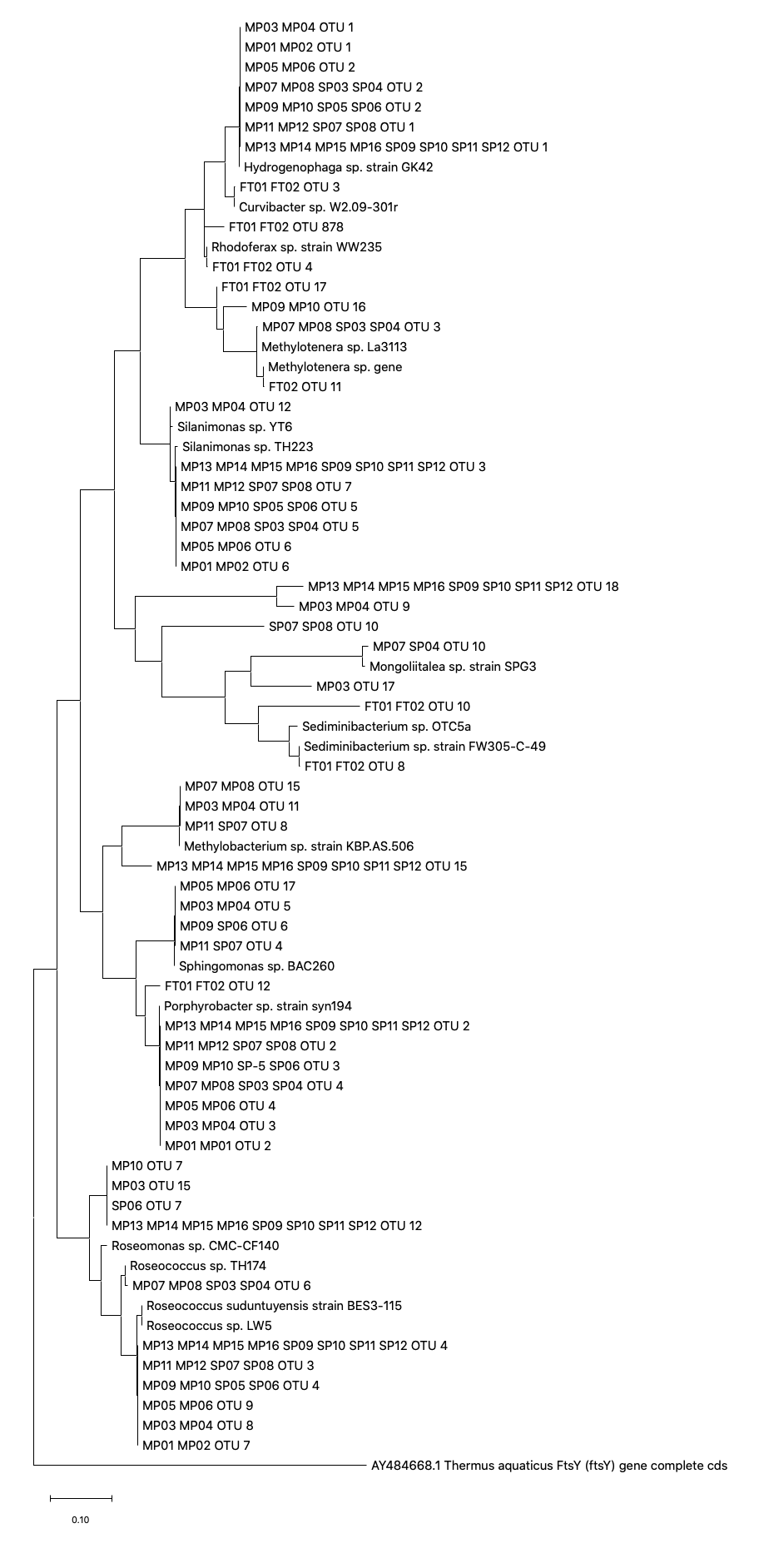


Supplementary Figure 1 Molecular Phylogenetic analysis by Maximum Likelihood method. The evolutionary history was inferred by using the Maximum Likelihood method based on the Tamura-Nei model (Tamura et al. 2004). The percentage of trees in which the associated taxa clustered together is shown next to the branches. Initial tree(s) for the heuristic search were obtained automatically by applying Neighbor-Join and BioNJ algorithms to a matrix of pairwise distances estimated using the Maximum Composite Likelihood (MCL) approach, and then selecting the topology with superior log likelihood value. The analysis involved 75 nucleotide sequences. All positions containing gaps and missing data were eliminated. There were a total of 194 positions in the final dataset. Evolutionary analyses were conducted in MEGA7 (Kumar et al., 2016)Bootstrap values (percentages) are given at the nodes.

Main Ponds

Subponds

Feeding tank

Supplementary Figure 2 Phylogenetic affiliations (closest known phyla) of microorganisms detected in Sellafield indoor pond (INP): feeding tank (FT), main ponds (MP) and subponds (SP) using Illumina sequencing with broad specificity primers for prokaryote 16S rRNA. Only the phyla that contained more than 1 % of the total number of sequences are shown.

Supplementary Table 2 Accession numbers deposited to the NCBI GenBank of the sequences identified on the INP

| **Uncultured Organisms** | |  |
| --- | --- | --- |
|  | **Accession Number** | **Bioproject** |
| Feeding tank (FT)  Main ponds (MP)  Subponds (SP) | SRP279469 | PRJNA660452 |
| **Subponds SP01 and SP02** | |  |
| **Pure Cultures** | **Accession Number** | **Submission Number** |
| *Cyclobacteriaceae bacterium*  *Cyclobacteriaceae bacterium*  *Algoriphagus* sp*.*  *Bacteroidetes bacterium*  *Aloriphagus* sp*.*  *Cyclobacteriaceae bacterium*  *Echinicola* sp.  *Cyclobacteriaceae bacterium* | MT585382  MT585383  MT585384  MT585385  MT585386  MT585387  MT585388  MT585389 | SUB7575476 |

Supplementary Table 3 Description of the media (selective and non-selective) used for microorganisms’ isolation

| **Media** | **Classification** | **Composition L^-1^** | **Final pH** | **Concentration %** | **Reference** |
| --- | --- | --- | --- | --- | --- |
| Minimal medium (M9) | Defined medium | Na_2_HPO_4_ 42.5 g  KH_2_PO_4_ 15 g  NH_4_Cl 5.0 g  MnCl_2_ 2.5 g  CuCl_2•_2H_2_O 43 mg  ZnCl_2_ 170 mg  CoCl_2_•6H_2_O 60 mg  Na_2_MoO_4_•2H_2_O 60mg | 7  10  11 | 10  50  100 | (Neidhart et al., 1974) |
| Luria Bertani (LB) | Complex Basal | Tryptone 10 g  Yeast extract 5 g  Sodium Chloride 10 g | 7  10  11 | 10  50  100 | (Sezonov et al., 2007) |
| Nutrient Agar for Aquiflexum (NA) | Complex Basal | KH_2_PO_4_ 0.3 g  Na_2_HPO_4_ 0.98 g  MgSO_4_ 0.10 g  NaCl 5 g  Yeast extract 5 g  Peptone 5 g  Agar 15 g | 7  10  11 | 10  50  100 | (Misal et al., 2013) |
| DL medium | Complex  Selective medium | NaHCO_3_ 2.5 g  Na_2_CO_3_ 5.0 g  NH_4_Cl 0.25 g  Na_2_H_2_PO_4_ 0.6 g  KCl 0.1 g  Vitamin mix 10 mL  Mineral mix 10 mL  Yeast extract 3.0 g  Peptone 4.0 g  Agar 10.0 g | 7  10  11 | 10  50  100 | (Lovley et al., 1984) |
| ZoBell | Complex  Selective medium | NaCl 19.45 g  MgCl_2_ 8.8 g  Na_2_SO_4_ 3.24 g  CaCl_2_ 1.8 g  C_6_H_5_FeO_7_ 0.1 g  Yeast extract 1.0 g  Peptone 5.0 g  Mineral mix 10 mL  Agar 15 g | 7  10  11 | 10  50  100 | (Brettar et al., 2004) |

Supplementary Table 4 Summary of the microorganisms identified on the different media used at a range of pH and media concentrations

| **Medium** | **pH** | **Growth** | **Organisms isolated and similarity percentage** | **Similarity (forward “F” and reverse “R”)** | **ID Most similar organisms (via BLASTn)** |
| --- | --- | --- | --- | --- | --- |
| **Minimal medium** | 7  10  11 | Growth was not detected at any concentration nor pH range | | | |
| **Zobell** | 7 | Detected at 10 % concentration | Strain S03: *Cyclobacteriaceae bacterium CUG 91308,* 93.5 % | F: 95 %  R: 92 % | 2483804 |
|  | 10 | Growth was not detected | | | |
|  | 11 | Detected at 50 % concentration | Strain S09: *Echinicola rosea strain JL3085,* 93.5 % | F: 96 %  R: 91 % | CP040106.1 |
| **Nutrient Agar for Aquiflexum**  **NA** | 7 | Detected at 10 % and 100 % concentration | Strain S01: Algoriphagus sp. XAY3209,91.5 %  Strain S05: *Algoriphagus* sp*. XAY3209,*91 % | F: 91 %  R: 92 %  F: 91 %  R: 91 % | 2007308  2007308 |
|  | 10 | Detected at 50 % concentration | Strain S02: *Echinicola* sp*. Strain CAU 1574,* 91 % | F: 94 %  R: 88 % | MN540912.1 |
|  | 11 | Not detected | | | |
| **DL** | 7 | Detected at 50 and 100 % concentration | Strain S08: *Echinicola* sp*. LN3S3,* 88 %  Strain S07: *Algoriphagus* sp*. R-36727,* 89.5 %  Strain S010: *Cyclobacteriaceae bacterium CUG 91308,* 85 % | F: 87 %  R:89 %  F: 89 %  R: 90 %  F: 86 %  R: 84 % | MN121190.1  885463  2483804 |
|  | 10 | Detected at 50 and 100% concentration | Strain S011: *Cyclobacteriaceae bacterium CUG 91308,* 91 %  Strain S06: *Algoriphagus* sp*. BAL344,* 91.5 % | F: 94 %  R: 88 %  F: 93 %  R:90 % | 2483804  1708148 |
|  | 11 | Detected at 50 and 100% concentration | Strain S04: *Bacteroidetes* sp*. BG31,* 91.5 % | F: 92 %  R:91 % | 1109254 |
| **LB** | 7  10  11 | Growth was not detected at any concentration nor pH range | | | |

Supplementary Table 5 Abundance of microorganisms detected by Sanger sequencing in the NGS Illumina MiSeq data

| **Sample** | **Organism identified by Sanger sequencing** | **Abundance detected by 16S NGS Illumina MiSeq** | **OTU** |
| --- | --- | --- | --- |
| **MP2_03 Oct17** | *Echinicola* sp | 0.28 % | OTU3 |
| **MP3_03 Oct17** | *Echinicola* sp | 0.39 % | OTU3 |
